# Comparison of the effect of different degrees of passive leg raising on the internal jugular vein cross-sectional area in patients before thoracic surgery

**DOI:** 10.1186/s12871-019-0751-5

**Published:** 2019-05-17

**Authors:** Shouyu Xie, Qimeng Yu, Tingting Li, Meiying Xu, Jingxiang Wu, Yanting Li

**Affiliations:** 1 Department of Anesthesiology, Shanghai Chest Hospital, Shanghai Jiao Tong University, No. 241 Huaihai Rd. West, Shanghai, China; 2 Department of Industrial Engineering and Management, School of Mechanical Engineering, Shanghai Jiao Tong University, Shanghai, China

**Keywords:** Thoracic surgery, Internal jugular vein, Cross-sectional area, Passive leg raising position

## Abstract

**Background:**

This study investigated the effect of different degrees of passive leg raising (PLR) on the internal jugular vein (IJV) cross-sectional area (CSA) and on the success rate of IJV cannulation in patients waiting for thoracic surgery, to analyze whether body mass index (BMI), gender, age, fasting time and preoperative rehydration have any impact on changes in the IJV CSA.

**Methods:**

Eighty-two patients scheduled for selective thoracic surgery were enrolled in this study. Patients were randomly assigned based on a computer-generated randomization sequence into 3 groups: 0, 30, and 50 degrees (*n* = 32, 25, and 25 patients, respectively). The right IJV CSA in the sequence of 0-degree (supine position), 30-degree and 50-degree PLR positions was recorded in all patients using an ultrasound probe. The relationship of BMI, gender, age, fasting time and preoperative rehydration to the IJV CSA was analyzed. Then, each patient was returned to a supine position. After waiting for at least 5 min, patients were placed in a PLR position at 0, 30, or 50 degrees, and then IJV cannulation was performed without ultrasound guidance. The success rate of IJV catheterization at different PLR angles was compared.

**Results:**

The average CSA of the right IJV in the supine position, 30-degree PLR position and 50-degree PLR position was 1.39 ± 0.63 cm^2^, 1.65 ± 0.73 cm^2^, and 1.68 ± 0.71 cm^2^, respectively. These results showed gradual increases in the IJV CSA of 18.5% (30-degree PLR) and 20.2% (50-degree PLR) when compared to that in the supine position (*P* = 0.045 and 0.025, respectively). However, only fasting time had a significant impact on the increase in the right IJV CSA at different PLR angles (*P* = 0.026). Other factors, such as BMI, gender, age and preoperative rehydration, had no significant effects. The success rates of IJV catheterization at angles of 0, 30 and 50 degrees were 84.3, 88 and 92%, respectively; however, there were no significant differences among the three groups (*P* = 0.674).

**Conclusions:**

PLR increases the CSA of the right IJV, especially for patients with long fasting times before thoracic surgery. The effect of the 30-degree PLR position is similar to that of the 50-degree PLR position. However, the success rate of right IJV catheterization was not enhanced in this study using landmark-guided puncture, even though the CSA of the right IJV was increased.

**Trial registration:**

Clinical trial registration number: ChiCTR1800015051. Date of registration: March 2018.

## Background

Surgical schedules are commonly overbooked in high-volume specialized hospitals in China. To reduce the turnaround times, some anesthetic procedures, such as internal jugular vein (IJV) cannulation, are performed in a preanesthesia room in advance for some cases. However, IJV cannulation can be very challenging in conscious, spontaneously breathing patients with potential hypovolemia because of overnight fasting and insufficient preoperative rehydration. The success rate of IJV catheterization may be influenced by many factors, including patient-specific factors (body mass index (BMI), gender and age), medical factors (catheterization technique, preoperative fasting time, preoperative rehydration) and body position [1]. Some of these factors affect the cross-sectional area (CSA) of the right IJV, which may be factors determining successful cannulation [2].

Although real-time ultrasound-guided IJV catheterization under general anesthesia has been strongly recommended to increase success rates and reduce complications [1, 3, 4], the reality is that this procedure is usually performed with an anatomical landmark-guided technique under local anesthesia in routine clinical practice in many hospitals in China. The widespread use of ultrasound-guided techniques has been restricted by the impracticality of manipulating an ultrasound machine and the lack of available equipment and trained personnel [3]. Therefore, it is necessary to find a practical method to improve the success rate of landmark-guided IJV catheterization in these conditions. Since catheterization is easier when the CSA of the IJV is large, various conditions have been investigated to maximize the IJV diameter, such as the Trendelenburg position, passive leg raising (PLR), Valsalva maneuver, abdominal binders, and positive end-expiratory pressure (PEEP) [5–11]. However, PEEP and abdominal binders are usually employed in anesthetized, mechanically ventilated patients, and the Valsalva maneuver and Trendelenburg position may be hazardous in some patients. Additionally, the Trendelenburg position cannot be used outside the operation room, while the PLR position may be a better option. Previous studies have shown that compared to the supine position, the 10-degree Trendelenburg and 40-degree PLR positions increase the size of the IJV to a similar extent (26% vs 23%, respectively) in mechanically ventilated patients [9]. However, whether a larger PLR angle increases the CSA of the IJV has not been thoroughly elucidated to date. Additionally, the size of the IJV may be related to other factors, such as BMI, gender, age, preoperative fasting time, and preoperative rehydration.

This study investigated the variation in the CSA of the IJV at different PLR angles in patients awaiting thoracic surgery (the primary endpoint) and assessed which of the abovementioned factors had an effect on the correlation between PLR angles and the IJV CSA. The secondary endpoint was to compare the success rate of IJV catheterization at different PLR angles using landmark-guided puncture.

## Methods

### Patients and data collection

The study protocol was approved by the Ethics Committee of the Shanghai Chest Hospital (permit no. KS1749) and registered as ChiCTR1800015051. The patients provided written informed consent before enrolment. Regular checks for plausibility and protocol adherence were performed according to good clinical practice (GCP) guidelines. The data safety and monitoring board (DSMB) for the study included two independent experts who were not part of the study group to ensure adherence to the study protocol, quality of data collection and processing and safety issues related to the study. All serious adverse events and all unexpected and related or possibly related adverse events were reported to the Internal Review Board of Shanghai Chest Hospital.

Eighty-two consecutive patients scheduled for elective lung cancer surgery between March 2018 and July 1st, 2018, were assessed for eligibility. All of the patients were fasted overnight and required central venous access as part of their treatment. The inclusion criteria were as follows: (1) American Society of Anesthesiologists (ASA) I or II patients scheduled to undergo thoracic surgery and (2) patients aged 18–80 years. The exclusion criteria were as follows: (1) patients who underwent thyroid surgery, (2) patients with major cardiovascular disease at baseline, (3) patients with end-stage renal disease requiring dialysis, (4) patients with a major infection (including but not limited to septicemia) and (5) patients with clinically important coagulopathy.

### Study design

**Fig. 1:**
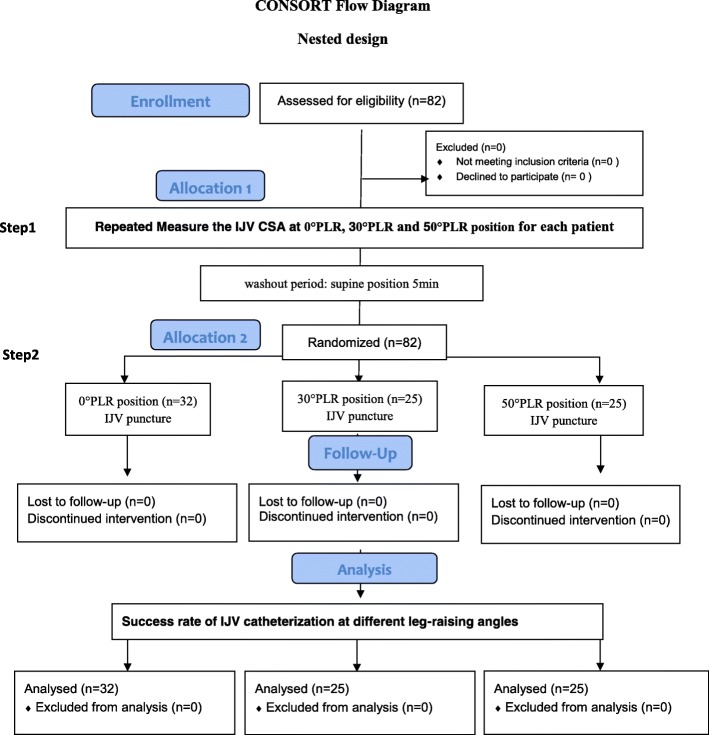
CONSORT flow diagram of the study

**Fig. 2:**
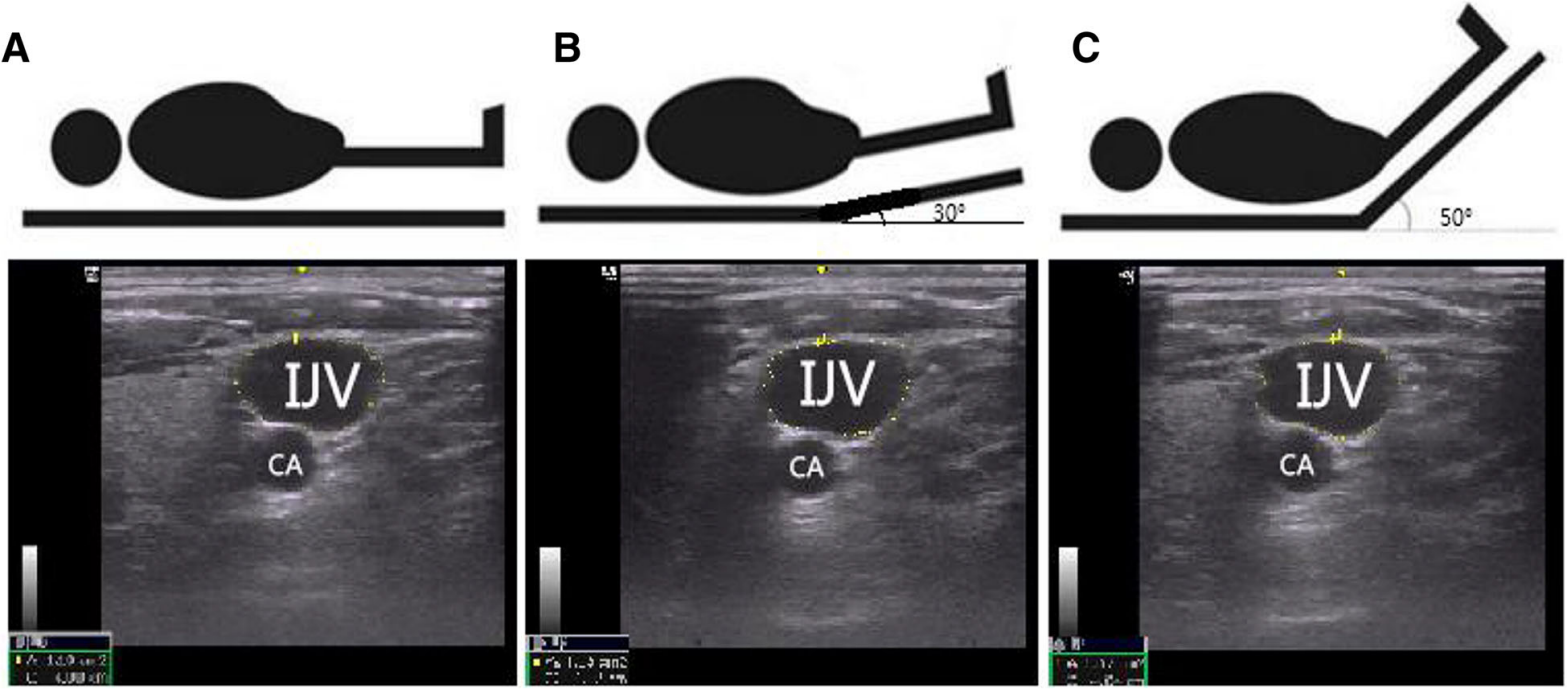
Schematic view and sonographic images of the IJV at different PLR positions. **a**. Supine position. **b**. 30-degree PLR position. **c**. 50-degree PLR position. IJV, internal jugular vein; CA, carotid artery

This prospective study had a nested study design that was divided into two steps (Fig. 1). We first measured the IJV CSA at 0, 30 and 50 degrees of PLR in each of the 82 patients with a repeated measures design. Then, after a return to steady state in the supine position, the same 82 patients were randomized to one of three subgroups with different PLR angles (0, 30 and 50 degrees) for IJV catheterization to calculate the success rate of IJV catheterization. The first step was a nonrandomized before-and-after self-controlled intervention trial with the aims of identifying the effect of PLR on the IJV CSA (primary endpoint) and determining whether BMI, age, gender, fasting time and preoperative rehydration were correlated to the IJV CSA. The second step of the study was a randomized controlled trial to evaluate the success rate of IJV catheterization using an anatomical landmark-guided technique at different PLR angles in patients who had completed the above evaluations. The patients were randomly assigned based on a computer-generated randomization sequence. Subjects with random numbers divisible by 3 were included in the first group, those with random numbers not divisible by 3 but with a remainder of 1 were included in the second group, and the remaining subjects were included in the third group. The patients were finally divided into 3 groups, namely, 0, 30, and 50 degrees of PLR (*n* = 32, 25, and 25 patients, respectively), which referred to the different PLR angles used during IJV catheterization (Figs. 1 & 2). The sample size for the first part was calculated based on 8 preliminary patients. The average difference in the CSA of the right IJV before and after 30-degree PLR was 0.2 cm^2^ (range: 1.2 ± 0.5 cm^2^ to 1.4 ± 0.6 cm^2^), with a standard deviation of 0.6. A minimum sample size of 73 patients was calculated to obtain 80% statistical power at a significance level of 0.05 (2-tailed). To allow for 10% loss during the study period, we intended to recruit a total of 82 patients. We did not calculate the minimum sample size for the second part of the study.

### Protocol

The right IJV was selected as the initial site of catheterization in all patients. In this study, ultrasonography with a 2-dimensional 8-MHz linear probe (LOGIQ e, GE Healthcare, Wauwatosa, WI, USA) was used. A roll appropriate for each patient was placed under their shoulders. With the head in a 15–30 degree contralateral position, sonographic images of the right IJV were recorded in sequence, as previously described [9], starting with the 0-degree PLR position (supine position), followed by the-30 degree and 50-degree PLR positions. Briefly, the probe was held perpendicular to the skin with minimal pressure without compressing the right IJV in a transverse axis at the apex of the triangle between the sternocleidomastoid muscle and the clavicle. The largest image of the vein was freeze-recorded at the end of the expiration to take measurements using electronic calipers and to evaluate the parameters on the ultrasound screen. All of these procedures were performed by the same anesthesiologist throughout the study. The PLR angles in these positions were adjusted using an angle meter. An inflatable pillow placed under the feet was used to adjust the position of the legs. Before recording the images, the patients were instructed to remain in the selected position for at least 1 min. After the vein images were recorded at each PLR angle, the patients once again returned to the supine position. The second part of the study was then initiated. After waiting for at least 5 min, the patients were positioned in the 0-, 30-, and 50-degree PLR positions according to how they had been randomly assigned as described above. IJV catheterization was performed by another anesthesiologist who was blinded to the size of the IJV, using the landmark-guided technique via a central approach in the anterior cervical triangle. The IJV was cannulated using a standard introducer needle with the aid of a seeker needle. The success rate of IJV puncture was recorded and compared. IJV cannulation was defined as a successful puncture, and catheterization was performed without the use of ultrasound. Failure to cannulate the IJV was defined as more than three attempts required to puncture the IJV with either a seeker needle or an introducer needle. Failure was also defined as puncture of the internal carotid artery with an introducer needle.

### Statistical analysis

SPSS 22.0 (IBM, Chicago, IL, USA) was used for data analysis. The normality of distribution was tested using the Kolmogorov-Smirnov test, and normally distributed data are expressed as the mean ± standard deviation. Data with a skewed distribution are expressed as medians (interquartile ranges). The effect of different PLR angles on the IJV CSA was analyzed using one-way ANOVA followed by Tukey’s honestly significant difference (HSD) post hoc test for multiple comparisons. Multivariate regression analysis was used to determine the association of BMI, gender, age, fasting time and preoperative rehydration to the IJV CSA. The correlation coefficient and confidence intervals (CIs) for the different variables and other statistics were calculated using Minitab 16 Software (Minitab, Inc., PA, USA) and Microsoft Excel (2016). Intergroup differences in the success rate of right IJV cannulation were assessed for statistical significance using the chi-square test. *P* < 0.05 was considered statistically significant.

## Results

### Baseline characteristics

Eighty-two patients were initially assessed for eligibility, and all completed a follow-up visit (Fig. 1). No patient was excluded from the study. The patients’ demographic characteristics are presented in Table 1. The average fasting time was 15.12 ± 3.75 h because of overnight fasting and late scheduled start times. The median preoperative rehydration volume was 250 (0, 500) ml (Table 1).

**Table 1:** Demographic data for the 82 patients

Age (years)	56.59 ± 11.28
Gender (male/female)	40 /43
BMI (kg/m^2^)	23.30 ± 3.58
Preoperative rehydration (ml)	250 (0, 500)
Fasting time (h)	15.12 ± 3.75

*BMI* Body mass index = weight(kg)/height(m)^2^

### Effect of different PLR angles on the IJV CSA

The average CSA of the right IJV in the supine position, 30-degree PLR position and 50-degree PLR position was 1.39 ± 0.63 cm^2^, 1.65 ± 0.73 cm^2^, and 1.68 ± 0.71 cm^2^, respectively (Table 2). The size of the IJV CSA increased significantly in the PLR positions (*P* = 0.017). The results in Table 3 showed a gradual increase in the IJV CSA of 18.5% (30-degree PLR position) and 20.2% (50-degree PLR position) compared to that in the supine position (*P* = 0.045 and 0.025, respectively). However, no significant difference was detected between the 30-degree and 50-degree PLR positions (*P* = 0.974).

**Table 2:** IJV CSA in different angles of leg raise positions

	N	CSA (cm^2^)	Kolmogorov-Smirnova	P-value
Supine	82	1.39 ± 0.63	0.191	0.017
30°PLR	82	1.65 ± 0.73	0.058	
50°PLR	82	1.68 ± 0.71	0.200	

*IJV* Internal jugular vein, *PLR* passive leg raising, *CSA* cross-sectional area, Kolmogorov-Smirnova test was used for normality checking; one-way ANOVA was used to test the difference

CSA Supine = 0.760–0.0040*BMI + 0.0455*Fasting time + 0.000253*Preoperative rehydration + 0.00294*Degree - 0.207*G (When Gender is Female, G = 1;When Gender is male, G = 0)

CSA 30°PLR = 0.715 + 0.0038*BMI + 0.0541*Fasting time + 0.000086*Preoperative rehydration + 0.00635*Degree - 0.289*G (When Gender is Female, G = 1;When Gender is male, G = 0)

CSA 50°PLR = 0.628 + 0.0068 BMI + 0.0528 Fasting time + 0.000146 Preoperative rehydration + 0.00569 Degree - 0.174*G (When Gender is Female, G = 1;When Gender is male, G = 0)

**Table 3:** Multiple Comparisons for IJV CSA in different angles of leg raise positions

CSA	CSA γ (%)	Mean Difference	95% CI	P-value
30°PLR – Supine	18.5	0.258	(0.005, 0.511)	0.045
50°PLR – Supine	20.2	0.282	(0.028, 0.535)	0.025
50°PLR – 30°PLR	3.1	0.023	(−0.230, 0.277)	0.974

*IJV* Internal jugular vein, *PLR* passive leg raising, *CSA* cross-sectional area, γ: The increment rate in the CSA is shown as a percentage; Tukey HSD was used for Post Hoc multiple comparisons

### Multivariate regression analysis of the relationship of BMI, gender, age, fasting time and preoperative rehydration to the IJV CSA

**Fig. 3:**
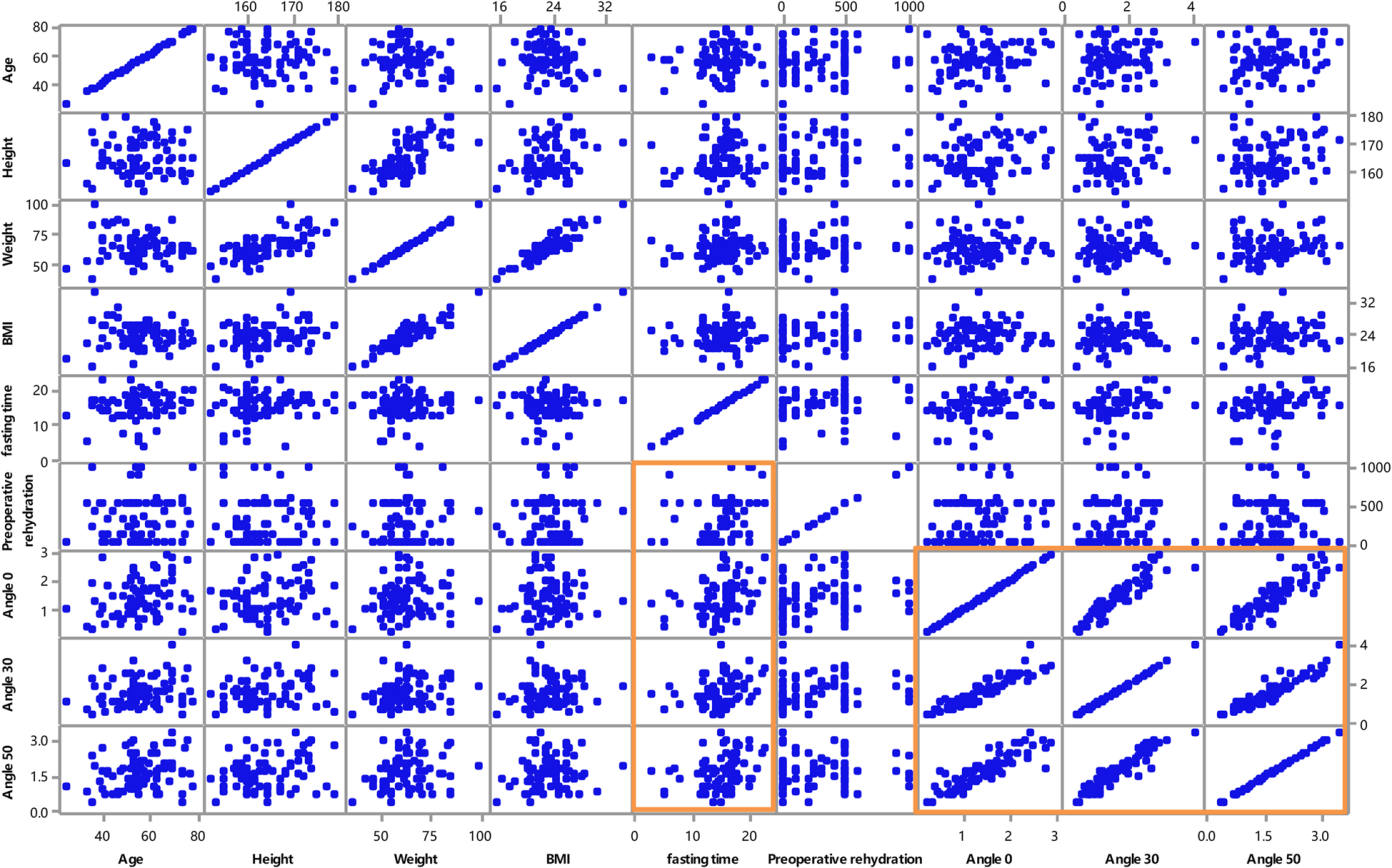
Overall correlation diagram of the factors related to the IJV CSA Scatter plots and a correlation matrix of patients’ weight, height, BMI, gender, age, fasting time, preoperative rehydration and CSA at 0 degrees, 30 degrees, and 50 degrees. Sections with significant correlations are identified using rectangles. IJV, internal jugular vein; CSA, cross-sectional area.

The correlation coefficients for weight, height, BMI, gender, age, fasting time and preoperative rehydration were calculated using Minitab Statistical Analysis, and the overall correlation results are shown in Fig. 3 and Table 4. The IJV CSA at the 0-degree, 30-degree, and 50-degree PLR positions showed a significant correlation (*P* < 0.05). The fasting time was correlated with the IJV CSA at different angles.

**Table 4:** Overall correlation of the factors

	Age	Height	Weight	BMI	fasting time	rehydration	Angle 0	Angle 30
Height	0.026							
Weight	−0.033	0.676^*^						
BMI	−0.091	0.257^*^	0.87^*^					
fasting time	0.158	0.199	0.148	0.064				
rehydration	0.054	−0.031	−0.01	0.037	0.472^*^			
Angle 0	0.195	0.179	0.156	0.094	0.347^*^	0.238^*^		
Angle 30	0.166	0.215	0.197	0.129	0.367^*^	0.200	0.939^*^	
Angle 50	0.216	0.176	0.162	0.103	0.366^*^	0.200	0.881^*^	0.937^*^

Correlation coefficient between factors. *P < 0.05, BMI, body mass index = weight(kg)/height(m)^2^

Since the height and weight did not show significant correlations as independent factors, the BMI was used to replace height and weight in the following regression analysis. A generalized linear model analysis was performed with fasting time as a covariate. We found that the PLR angle had a significant effect on the CSA (*P* < 0.05). When the fasting time was the same, the CSA obtained in the 30-degree PLR position was the largest. The longer the fasting time was, the larger the angle needed to obtain the same CSA. The estimated regression coefficients and *P*-values of the five factors in relation to the CSA are shown in Table 5.

**Table 5:** Multivariate regression analysis of the BMI, sex, age, fasting time and preoperative rehydration with CSA

factors	Angle 0		Angle 30		Angle 50	
	estimate	P-value	estimate	P-value	estimate	P-value
sex	−0.103	0.553	−0.148	0.457	0.010	0.960
age	0.008	0.215	0.008	0.305	0.012	0.105
BMI	0.004	0.605	0.008	0.349	0.010	0.212
Fasting time	0.043	**0.031** ^*^	0.053	**0.020** ^*^	0.049	**0.026** ^*^
preoperative rehydration	0.000	0.345	0.000	0.802	0.000	0.631

*P < 0.05, R-square = 13.6% *P* < 0.0001 when analysed with general line model

### Success rate of IJV cannulation

No significant difference in the success rate of IJV cannulation at different angles was detected (*P* = 0.674, Pearson chi-square test) (Table 6). Puncture of the internal carotid artery with the introducer needle did not occur in these cases.

**Table 6:** success rate of IJV puncturing

	Angle 0	Angle 30	Angle 50	P-value
success rate	84.3%	88%	92%	0.674

## Discussion

The main finding of the current study showed that PLR remarkably increased the IJV CSA in patients waiting for thoracic surgery with an increment rate of 18.5% (30-degree PLR position) and 20.2% (50-degree PLR position) compared to that in the supine position, whereas no significant difference was found between the two angles. BMI, gender, age and preoperative rehydration had no significant effect on the right IJV CSA in relation to leg raising. Only the fasting time had a significant impact (*P* = 0.026). The longer the fasting time was, the larger the angle needed to obtain the same IJV CSA. However, there were no significant differences in the success rate of IJV cannulation when using landmark-guided puncture at different PLR angles, even as the IJV CSA increased.

As patients awaiting thoracic surgery in a preanesthesia room may have experienced prolonged overnight fasting times, IJV catheterization can be very challenging due to potential hypovolemia because of insufficient preoperative rehydration. Therefore, the purpose of this study was to explore a simple and safe method to increase the CSA of the IJV in conscious patients with spontaneous breathing to improve the success rate of IJV puncture in patients waiting for thoracic surgery in the preparation area and to test the possible influencing factors. It was found that with the increase in the angle of the PLR position, the CSA of the IJV was indeed increased. However, it was interesting that although the area increased, the success rate of the puncture was not significantly improved if we still used the landmark-guided puncture. As real-time ultrasound-guided puncture is still not in the mainstream in our clinical practice for many practical reasons, the results of this study further suggest that even though the PLR maneuver increases the area of the IJV, the landmark-guided puncture success rate is still not significantly increased, once again stressing the necessity and advantage of ultrasound-guided puncture. Another interesting finding of this study was that although the leg raising angle was raised from 30 to 50 degrees, the increase in the area of the IJV was no longer significant, suggesting that 30 degrees may be sufficient in the PLR maneuver. In addition, among the many factors affecting the CSA of the IJV, only the length of fasting time plays a significant role, which is less related to BMI, gender, age, etc. The lack of a significant effect of preoperative fluid rehydration may indicate that the current strategy of fluid rehydration may be insufficient for the loss of body fluids caused by fasting.

Many factors influence the diameter of the IJV and IJV cannulation. The mean diameter of the right IJV has been reported to range from 10.6 mm to 13.5 mm [12, 13], but it can be less than 5 mm in 13–18% of cases due to anatomical variation, the patient’s blood volume, positioning, head rotation, and other conditions [5, 14]. The success rate of IJV catheterization may be influenced by other factors, including patient-specific factors (BMI, gender and age), medical factors (catheterization technique, preoperative fasting time, preoperative rehydration) and body position [1]. These factors affect the CSA of the right IJV and may thereby determine the level of success of cannulation. Trendelenburg or PLR positions were the two simplest approaches to increase IJV size. As the Trendelenburg position cannot be used outside the operation room and the PLR position is easier to apply than the Trendelenburg position [9], we chose the PLR position for evaluation.

The PLR position is usually used for patients with hypovolemic shock to redistribute the blood volume from the lower extremities to the head and neck circulation or to guide fluid administration [15–18]. It was reported that PLR could increase the IJV diameter. Gok et al. reported that the 10-degree Trendelenburg and 40-degree PLR positions increased the size of the IJV to a similar extent [9]. Our study adds to the current literature published by Gok et al., whose findings apply to patients who are mechanically ventilated. Mechanical ventilation, with positive pressure, can increase the volume of the IJV by increasing intrathoracic pressure, decrease blood flow to the heart and increase the IJV CSA under general anesthesia [6]. In this study, patients were not on mechanical ventilation. Therefore, in this case, PLR can be a useful maneuver to increase the IJV CSA in nonmechanically intubated patients. A previous study evaluating the effects of PLR and low head height on the CSAs of the IJV and subclavian vein (SCV) showed that PLR increases the CSA of the IJV but has little effect on the SCV CSA [19]. The CSA of the IJV seems to be more affected by gravity than that of the SCV [19, 20]. The researchers reported that the mean CSA of the IJV in awake adults was 1.12 ± 0.57 cm^2^ in the supine position, 1.66 ± 0.67 cm^2^ in the Trendelenburg position, 0.38 ± 0.23 cm^2^ in the reverse Trendelenburg position, and 1.40 ± 0.64 cm^2^ during 50-degree PLR [19]. The mean CSA of the IJV in our study was 1.65 ± 0.73 cm^2^ at 30 degrees and 1.68 ± 0.71 cm^2^ at 50 degrees vs 1.39 ± 0.63 cm^2^ in the supine position. These trends are consistent with their results. However, the subjects in the current trial were all patients, whereas the former study used normal, healthy people [19]. The fasting time was also different. The patients in this experiment fasted for at least 8 h, while no one in the former study fasted [19], a factor that may have affected the experimental results. No person in the former study underwent IJV catheterization because it was only an observational study [19]. Each patient in the present investigation underwent IJV catheterization to study the effects of different PLR angles on the IJV CSA in patients undergoing thoracic surgery.

Our study revealed that increasing the PLR angle may increase the CSA of the IJV. However, the CSA of the IJV did not increase in every patient when the PLR angle was increased from 30 to 50 degrees. This is an interesting finding, although the mechanism is unclear. Similar conditions have been reported in other studies in which a lesser or limited increase was found in the CSA of the IJV in the increased Trendelenburg position [6, 21, 22]. Nassar et al. even reported a decrease in the CSA in nine of fifty patients in a 15° Trendelenburg position [22]. Likely explanations for this finding include the following: (1) a compensatory mechanism in the human body may be at play; once the IJV is distended, it becomes progressively less distensible, which decreases the degree of filling of the vein with time. (2) The PLR position may heighten sympathetic output in some subjects, augmenting venous tone and reducing the CSA. The jugular veins are not simply collapsible tubes but rather possess actively contracting walls [23]. (3) A much less likely explanation for a reduced CSA upon assuming the PLR position is the redistribution of blood volume from the lower extremities to the inferior vena cava rather than to the IJV.

In the present study, fasting time, but not preoperative rehydration, was significantly correlated with the CSA of the IJV, suggesting that preoperative rehydration measures were insufficient. The suggestion to shorten the fasting time of a patient as much as possible for enhanced recovery after surgery (ERAS) was published in the latest edition of the ASA guidelines on January 3, 2017 [24]. It has now been well established that a time period of 6 h between the last solid meal and induction of anesthesia or 2 h between the last intake of clear fluids and induction of anesthesia is safe in the majority of patients [24]. However, actual difficulties could contribute to the lack of widespread use of reduced preoperative fasting protocols in daily practice, such as flexible daily surgical scheduling subject to last-minute changes [25]. The ‘nil by mouth from midnight’ rule before elective surgery is still used in patients [25]. The average fasting time was 15.12 ± 3.75 h in the present study, which was quite long because of overnight fasting and late scheduled start times. Even if the patients were rehydrated with approximately 250 ml fluid before IJV cannulation, the loss of capacity after long fasting cannot be completely compensated for by preoperative rehydration [24]. Our previously unpublished data also revealed that the success rate of IJV catheterization in the morning is higher than that in the afternoon, even if the patients are preoperatively rehydrated.

Although arterial puncture rates between 4.3 and 25% have been reported [26], puncture of the internal carotid artery with an introducer needle did not occur in our patients. However, we need to be aware that carotid artery injury is one of the most serious complications of IJV catheterization [27]. In this trial, no significant difference in the success rate of cannulation in the three positions was detected. Thus, the increase in the IJV CSA contributes little to the success rate of cannulation if landmark-guided puncture is used, which may be due to anatomic variation in the IJV.

There were several limitations in this study. (1) The second part of the study in which the success rate of IJV catheterization was evaluated may have been underpowered because the sample size was not calculated and because there was insufficient time between parts one and two of the study (> 5 min). Therefore, we mainly used a descriptive analysis for the second part. Nevertheless, we still claim that our study has strong statistical power with IJV CSA examinations, validated measurement tools and reliable outcome assessments to generate conclusions. (2) The anesthesiologist who measured the CSA of the IJV was not blinded to the PLR position. This anesthesiologist could have influenced the measurements (e.g., measuring a larger CSA rather than being conservative during measurement) to show some benefit of the PLR position on the CSA. This approach could have introduced major bias. We asked the anesthesiologist who performed the evaluation to follow the standard operation protocol described in the method to avoid measurement deviation to the greatest extent. We also used the increment rate in the CSA to avoid bias. (3) Blinding of the doctors involved in this study was inapplicable because it is relatively easy to determine a patient’s leg position. (4) We defined turning of the head as an angle of 15–30 degrees to avoid a possible decrease in venous diameter due to excessive turning of the head; however, it is not possible to precisely measure this angle. We put a disposable mask on the contralateral side of the neck to aid in judging the angle to avoid excessive turning of the head.

## Conclusions

The present study demonstrated that PLR increases the CSA of the right IJV, especially in patients with long fasting times (more than 15 h of overnight fasting) before thoracic surgery. The longer the fasting time is, the larger the angle needed to obtain the same CSA. The effect of the 30-degree PLR position is similar to that of the 50-degree PLR position. However, the success rate of the right IJV catheterization was not significantly different with the use of the landmark-guided puncture, even in cases in which the CSA of the right IJV increased.

## Abbreviations

ASA: American Society of Anesthesiologists
BMI: Body mass index
CSA: Cross-sectional area
CVC: Central venous cannulation
IJV: Internal jugular vein
ICU: Intensive care unit
PLR: Passive leg raising
US: Ultrasonography
